# A case-control association study of *NRXN1 *polymorphisms with schizophrenia in Chinese Han population

**DOI:** 10.1186/1744-9081-7-7

**Published:** 2011-04-11

**Authors:** Weihua Yue, Yongfeng Yang, Yanling Zhang, Tianlan Lu, Xiaofeng Hu, Lifang Wang, Yanyan Ruan, Luxian Lv, Dai Zhang

**Affiliations:** 1 Institute of Mental Health, Peking University; Beijing, 100191, PR China; 2 Key Laboratory for Mental Health, Ministry of Health, Beijing, 100191, PR China; 3 Department of Psychiatry of the Second Affiliated Hospital of Xinxiang Medical University, Xinxiang, 453002, PR China; 4 Henan Mental Hospital, Henan Key Lab of Biological Psychiatry, Xinxiang, 453002, PR China

**Keywords:** Schizophrenia, Neurexin-1 (NRXN1), Haplotype, age-at-onset

## Abstract

**Background:**

Recent research has implicated that mutations in the neurexin-1 (*NRXN1*) gene on chromosome 2p16.3 might play a role in schizophrenia, autism, and nicotine dependence. In order to explore the association of *NRXN1 *polymorphisms with schizophrenia, we made a case-control association study in Chinese Han population.

**Methods:**

We examined six tag single nucleotide polymorphisms (SNPs) spanning 116.7 kb of *NRXN1 *in 768 schizophrenic patients and 738 healthy control subjects. The association of *NRXN1 *polymorphisms with schizophrenia and the age-at-onset of this disease were explored.

**Results:**

Our results showed that four SNPs of *NRXN1 *gene were significantly associated with schizophrenia (rs10490168: G > A, *p *= 0.017; rs2024513: A > G, *p *= 0.006; rs13382584: T > C, *p *= 0.009; and rs1558852: G > A, *p *= 0.031). Furthermore, the association of SNP rs2024513 with schizophrenia remained significance after the Bonferroni correction. Haplotypes consisting of above six SNPs also showed significantly associated with schizophrenia (global chi-square = 14.725, *p *= 0.022). A protective haplotype AGTGCA remained associated with schizophrenia, even after 10,000 permutation tests (empirical *p*-value = 0.043). However, we did not find any association with age-at-onset of schizophrenia with *NRXN1 *polymorphisms.

**Conclusions:**

Our findings suggest that *NRXN1 *might represent a major susceptibility gene for schizophrenia in Chinese Han population.

## Background

Schizophrenia is a common psychiatric disorder characterized by profound disturbances of thinking, emotion, and social functioning. It affects approximately 1% of the worldwide population and accounts for about 2.5% of healthcare costs [1]. Aberrant synaptic connectivity is a prominent feature of schizophrenia's neuropathology [2]. Recently, several groups have identified schizophrenic patients who showed microdeletions disrupting the promoter and N-terminal encoding exons of neurexin 1 (*NRXN1*) alpha [3-5]. Kirov et al. have found a deletion disrupting the *NRXN1 *gene at 2p16.3 in a mother and two affected siblings from one family, and identical twins concordant for child-onset schizophrenia, respectively [3-5]. Furthermore, copy number variations (CNVs) in *NRXN1 *gene have also previously been implicated in autism, mental retardation and nicotine dependence [6-11].

Neurexins, including NRXN1, NRXN2, NRXN3, are cell-surface receptors that bind neuroligins (NLGNs) and form a Ca^2+^-dependent neurexin/neuroligin complex at synapses in the central nervous system [12,13]. The NRXN - NLGN complex plays an important role during the GABAergic and glutamatergic neurotransmission [12]. There is prevailing evidence implicating both neurexins and neuroligins as primary factors in neuropsychiatric disorders [3-11].

All these indicate that *NRXN1 *might be a strong candidate gene for schizophrenia [3-6]. Resequencing has been applied to identify the rare mutations of this gene, but the linkage and association studies of *NRXN1 *polymorphisms in Chinese Han population remained still insufficient. In this study, we attempted to investigate the association between the *NRXN1 *gene polymorphisms and schizophrenia and age-at-onset of this disease in 768 patients with schizophrenia and 738 healthy control subjects of Chinese Han population.

## Methods

Our study sample consisted of 768 patients with schizophrenia (360 men and 408 women; mean age: 33.5 ± 8.7 years) and 738 healthy controls (358 men and 380 women; mean age: 32.2 ± 6.4 years). All of the participants were Chinese Han descendants. The patients were recruited from the Institute of Mental Health, Peking University, China. The consensus diagnoses were made by at least two experienced psychiatrists according to the Diagnosis and statistic manual of mental disorders, 4^th ^edition (DSM-IV) criteria for schizophrenia. The age of clinical symptoms onset (12~53 years, mean 23.8 ± 7.8 years) was retrospectively estimated as the time of emergence of any schizophrenic symptoms according to the DSM-IV. None had severe medical complications. Healthy controls were recruited from communities with simple non-structured interview performed by psychiatrists, who excluded individuals with history of mental health and neurological diseases. The control subjects live in the same area with patients, and were group-matched to patients by gender, age, and ethnicity. The objectives and procedures of the study were explained to all participants and written informed consent was obtained. Research ethics committee approval was obtained from the Ethical Committee of Institute of Mental Health, Peking University.

Peripheral blood samples were collected from all subjects. Genomic DNA was extracted using the Qiagen QIAamp DNA Mini Kit. Considering Kirov et al.'s report that micordeletions spanning the promoter and the first exon of the *NRXN1 *gene might increase the risk of developing schizophrenia, we selected six tag single nucleotide polymorphisms (tagSNPs) locating the promoter and the first exon of this gene, according to the dbSNP database (http://www.ncbi.nlm.nih.gov/SNP) [3-5]. All of the six tagSNPs were genotyped with the TaqMan genotyping methods according to the manufacturer's protocol, where allelic discrimination and analysis were performed using an ABI Prism 7300 Sequence Detection System (Applied Biosystems, CA). The information of ABI Taqman assays and context sequences of six SNPs are given in Table 1. The standard TaqMan^® ^SNP genotyping assay protocol was performed for polymerase chain reactions (PCRs), which contained 10 ng of DNA, 5 μL of 2 ×TaqMan Universal PCR Master Mix (Applied Biosystems, Foster City, CA), 0.5 μL of 20 × SNP Genotyping Assay Mix (Applied Biosystems, Foster City, CA), and 4.5 μL of water, for a total volume of 10 μL. The conditions of PCR amplification were as follows: 1 enzyme activation step at 95.0°C for 10 min, and 40 alternating cycles of denaturation at 92.0° C for 15 s and re-annealing and extension at 60.0°C for 60 s. To evaluate the quality of genotyping, 5% of the samples were randomly selected and re-genotyped, and the genotyping consistency rate was more than 98%.

**Table 1:** Information of TaqMan assays and context sequences of 6 SNPs examinaed

ID	SNPs	Position	Location	Context Sequence ([VIC/FAM])
1	rs10490168	50988978	intron	TGAATTGATAAAACAGTAACACTGA[C/T]AAAAATACATACACACATTCACACAC
2	rs2024513	51005523	intron	GAAGTGTTCTTTCTTAGATACATGA[A/G]GTCTTGGTAACCTTAATGGCTATTT
3	rs10174398	51049105	intron	ACCAGTCCACCTCTATCTGTACCCA[C/T]GCTCTCTCCATATAATGTTCTTCTC
4	rs10195460	51082210	intron	CTAAAGGGAGATCAATTAAGCATAC[A/G]TTTTTCCACCTTTTAAAATAATACT
5	rs13382584	51100798	intron	TCTTTACAAATGTAACCACCACCCA[C/T]ATCATGCCCAATGCTCCATTGTTTT
6	rs1558852	51105641	intron	TTAATACATGATCTGTATTGGGAGA[A/G]TCATCCATTCTCAATTAATTATTCA

The statistic power of our sample size was calculated by the genetic power calculator (GPC, http://pngu.mgh.harvard.edu/~purcell/gpc/cc2.html) [14]. Deviation of the genotype counts from the Hardy-Weinberg equilibrium was tested using a chi-square goodness-of-fit test. The pairwise linkage disequilibrium (LD) analysis was applied to detect the inter-marker relationship, using *D'*-values. The case-control association analysis was performed by the Haploview version 4.1 (http://www.broad.mit.edu/mpg/haploview/), a powerful software platform for computing single locus and multi-marker haplotype association tests [15]. Bonferroni corrections for multiple tests were carried out to exclude type I errors. For the haplotype analyses, 10,000 times of permutation tests were performed to control false positive results. The LD plot of 6 SNPs at the 5'UTR of *NRXN1 *gene and its regional plots across this gene were constructed by using the Haploview v4.1 software in 738 healthy control subjects. To indicate the relative position of above 6 SNPs and the other SNPs across the *NRXN1 *gene download from the HapMap stage II dataset, we then further constructed the regional LD plots using the LocusZoom (http://csg.sph.umich.edu/locuszoom/) [16]. Associations between age-at-onset and different genotype carriers were determined by one-way ANOVA tests with the SPSS 13.0. Considering that the mean age of control group was younger than that of case group, we implemented the logistic regression analysis, with diagnosis as dependent variable, and 6 tagSNPs and age at enrolled of subjects as independent variables. Results were considered significant at two-tailed *P *< 0.05.

## Results

Six SNPs in the *NRXN1 *gene were genotyped in 768 schizophrenia patients and 738 healthy control subjects of Chinese Han population. The size of our sample was sufficient to detect a significant difference with a power of more than 90% assuming an odds ratio (OR) values of AA as 1.7 with a minor allele frequency of 0.1. The genotype distributions of the six SNPs for patients and controls were in Hardy-Weinberg equilibrium (Table 2). Allele and genotype frequencies of the six SNPs between patients and controls are shown in Table 2. Significant differences were found in allele frequencies between patients and controls at four SNPs, rs10490168 (G > A, *p *= 0.017), rs2024513 (A > G, *p *= 0.006), rs13382584 (T > C, *p *= 0.009), and rs1558852 (G > A, *p *= 0.031). However, only the difference in the allele frequencies of rs2024513 remained significance after the Bonferroni correction. For control the confounding effects of age of subjects, we used the logistic regression to identify whether the age influence the marker-diagnosis effects. However, the age of subjects when they were enrolled in the current study did not showed any significant confounding effects on maker-diagnosis association (*p *= 0.058).

**Table 2:** Comparison of genotype and allele frequencies of six SNPs at the *NRXN1 *gene between schizophrenic patients and healthy control subjects

Makers	Genotype N (Freq.)			Chi-square (df = 2)	*p- *value	HWEP	Allele N (Freq.)		Chi-square (df = 1)	*p*- value	OR (95% CI)
rs10490168	AA	AG	GG				A	G			
Cases	10(0.013)	173(0.225)	585(0.762)	6.049	**0.048**	0.485	193(0.126)	1343(0.874)	5.677	**0.017**	0.78(0.63-0.96)
Controls	13(0.018)	204(0.276)	521(0.706)			0.168	230(0.156)	1246(0.844)			
rs2024513	AA	AG	GG				A	G			
Cases	544(0.708)	206(0.268)	18(0.023)	7.521	**0.023**	0.772	1294(0.842)	242(0.158)	7.327	**0.006**	1.30(1.07-1.56)
Controls	480(0.650)	228(0.309)	30(0.041)			0.655	1188(0.805)	288(0.195)			
rs10174398	CC	CT	TT				C	T			
Cases	276(0.359)	359(0.467)	133(0.173)	0.727	0.695	0.382	911(0.593)	625(0.407)	1.064	0.397	1.06(0.92-1.23)
Controls	254(0.344)	345(0.467)	139(0.188)			0.256	853(0.578)	623(0.422)			
rs10195460	AA	AG	GG				A	G			
Cases	61(0.079)	318(0.414)	389(0.507)	1.197	0.549	0.721	440(0.286)	1096(0.714)	0.972	0.323	0.92(0.79-1.08)
Controls	62(0.084)	323(0.438)	353(0.478)			0.321	447(0.303)	1029(0.697)			
rs13382584	CC	CT	TT				C	T			
Cases	14(0.018)	200(0.260)	554(0.721)	7.075	**0.029**	0.404	228(0.148)	1308(0.852)	6.821	**0.009**	0.77(0.64-0.94)
Controls	23(0.031)	225(0.305)	489(0.664)			0.638	271(0.184)	1203(0.816)			
rs1558852	AA	AG	GG				A	G			
Cases	234(0.305)	383(0.499)	151(0.197)	4.666	0.097	0.799	851(0.554)	685(0.446)	4.627	**0.031**	0.85(0.74-0.99)
Controls	259(0.351)	357(0.484)	122(0.165)			0.956	875(0.593)	601(0.407)			

Freq., frequency; HWEP: the p-value for Hardy-Weinberg equilibrium tests; OR, odds ratio; CI, confidence interval.

**Figure 1:**
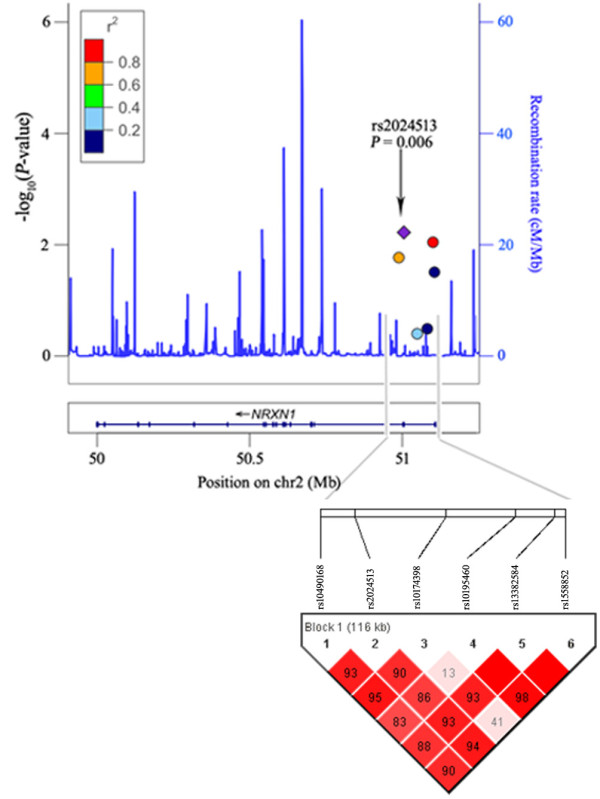
**Linkage disequilibrium plot consist of 6 SNPs at the *NRXN1 *gene and its regional plots**. Pairwise linkage disequilibrium (LD) was computed for all possible combinations of the six SNPs using *D' *values. Six SNPs were plotted with the -log_10_(*P*-values) in the *NRXN1 *genomic position (in the University of California Santa Cruz March 2006 human reference sequence, hg18). Estimated recombination rates of SNPs in *NRXN1 *regions (taken from HapMap Japanese in Tokyo (JPT) + Chinese Han in Beijing (CHB)) are plotted to reflect the local LD structure. The index association SNP is represented by a diamond. Color of the remaining SNPs (circles) indicates LD with the index SNP based on pairwise *r*^*2 *^values from HapMap data.

To further analyze the haplotype structure in our sample, we computed pairwise LD for all possible combinations of the six SNPs using *D' *and *r*^*2 *^values. Figure 1 showed the LD plot of constructed by the six SNPs locating at the 5'UTR of *NRXN1 *and regional LD plots across the *NRXN1 *gene. Haplotypes consisting of above six SNPs also showed significantly associated with schizophrenia (global chi-square = 14.725, *p *= 0.022). A protective haplotype AGTGCA remained associated with schizophrenia, even after 10,000 permutation tests (empirical *p*-value = 0.043).

To explore the potential association of age-at-onset of schizophrenia with *NRXN1 *polymorphisms, we carried out association analysis tests using the six SNPs at *NRXN1 *gene. However, the results of one-way ANOVA tests revealed there was no association of age-at-onset of schizophrenia with six SNPs at *NRXN1 *gene (*p *> 0.05) (data not shown). Furthermore, we also did not find the association between *NRXN1 *polymorphisms and early-onset schizophrenia.

## Discussion

Neurexins, including NRXN1 and other members of the neurexin family, are synaptic neuronal adhesion molecules. NRXNs could bind neuroligins (NLGNs) to form a Ca^2+ ^- dependent NRXN - NLGN synpatic complex in the central nervous system. This NRXN - NLGN complex is required for neurotransmission at synapses and is involved in the presynaptic and postsynaptic differentiation of GABAergic and glutamatergic synapse [12,17-20]. The de novo alterations of the *NRXN1 *gene and their roles in the pathogenesis of autism and schizophrenia have been reported by some studies [4,21,22]. Based on these findings, we hypothesized that the *NRXN1 *might be a strong candidate gene for schizophrenia. However, there has been no further evidence of association between *NRXN1 *polymorphisms and schizophrenia in Chinese Han population. In the present study, we investigated the association of *NRXN1 *polymorphisms and schizophrenia in 768 cases and 738 healthy control subjects of Chinese Han population. We detected six tagSNPs of *NRXN1 *gene in our samples. Significant differences were found in allele frequencies between patients and controls at four SNPs (rs10490168, rs2024513, rs13382584, rs1558852). However, only the difference in the allele frequencies of rs2024513 remained significance after the stringent Bonferroni multiple-test correction. Further association analysis of haplotypes consist of six SNPs at the *NRXN1 *gene with schizophrenia were performed. Three haplotypes at the *NRXN1 *gene were found to be associated with schizophrenia. The protective haplotype AGTGCA consist of above 6 SNPs remained significantly associated with schizophrenia, even after Bonferroni correction (Table 3).

**Table 3:** Comparison of haplotype frequencies at the *NRXN1 *gene between schizophrenic patients and healthy control subjects

Haplotype	Case (Freq.)	Control (Freq.)	Chi - square	*p *- value	OR (95%CI)
AGTGCA	167.76(0.109)	207.23(0.141)	6.770	0.009	0.75(0.60-0.93)
GACATA	208.91(0.136)	233.02(0.158)	2.904	0.088	0.84(0.68-1.03)
GACGTA	164.41(0.107)	134.86(0.091)	2.072	0.150	1.19(0.94-1.52)
GACGTG	510.60(0.332)	453.08(0.307)	2.287	0.130	1.13(0.97-1.32)
GATATA	214.77(0.140)	192.85(0.131)	0.542	0.461	1.08(0.88-1.34)
GATGTG	157.26(0.102)	135.07(0.092)	1.018	0.312	1.13(0.89-1.44)
GGTGCA	39.15(0.025)	49.39(0.034)	1.682	0.194	0.76(0.49-1.16)
Global			14.725	0.022	

Freq., frequency; OR: odds ratio; CI: confidence interval

By microarray analysis in 2,977 European patients with schizophrenia and 33,746 European controls, Rujescu et al. identified 66 deletions and 5 duplications in the *NRXN1 *gene: 12 deletions and 2 duplications occurred in schizophrenic patients (0.47%), compared to 49 and 3 in controls (0.15%) [5]. There was no common breakpoint, and the CNVs varied from 18 to 420 kb. No CNVs were found in the *NRXN2 *or *NRXN3 *genes. By restricting the association analysis to CNVs that disrupted exons, they identified a significant association with a high odds ratio (0.24% cases *vs*. 0.015% controls, *p *= 0.0027, OR = 8.97). Rujescu et al suggested that *NRXN1 *deletions affecting exons may confer risk of schizophrenia [5]. Several previous studies have suggested that the *NRXN1 *gene might be the susceptibility gene of schizophrenia and other neurodevelopmental disorders.

Kirov et al.'s reports CNVs in *NRXN1 *gene implicated with schizophrenia and other mental disorders mostly are the rare polymorphisms [3]. Several other published literatures also supported that the rare CNVs in *NRXN1 *gene may be associated with schizophrenia and other mental disorders. However, schizophrenia has been regarded as a complex disease, so there might be many susceptibility genes associated with this disease. To explain the association of polymorphisms with complex disorders, we have two models, the 'common disease/common variant' (CDCV) and 'common disease/rare variant' (CDRV) model [23]. The CDCV model of schizophrenia suggests that relative common (often > 5%) genetic variants in population might confer minor or modest risk (i.e., OR = 1.1-1.5). On the other hand, the CDRV model postulates that complex traits might derive from many rare mutations for individuals (usually <1%) but with relative strong effects (i.e., perhaps OR > 10). In the present study, we want to discuss that common SNPs in *NRXN1 *gene, other than rare CNVs, might also be associated with schizophrenia in Chinese Han population. We focused on the association of six SNPs spanning a 117.6 kb region locating in an intron near the *NRXN1 *5'UTR with schizophrenia. And our data of both SNPs and haplotypes showed that the *NRXN1 *might be a susceptibility gene of schizophrenia in Chinese Han population.

Walsh et al. reported that novel deletions and duplications of *NRXN1 *gene presented more frequently cases (15%) and young-onset cases (20%) with schizophrenia than those in controls (5%) [4]. In the present study, we did not do any association analyses of child-onset schizophreia with *NRXN1 *polymorphisms, since we have only five patients with age-at-onset less than 12 years old in our 768 patients with schizophrenia. Although we did association tests between early-onset (<18 years old) schizophrenia and *NRXN1 *polymorphisms, we did not find an association. In all, we did not find any association of *NRXN1 *polymorphisms with age-at-onset of schizophrenia. This may be partly explained by the ethnic difference across various populations. The diversity of genotype and allele distributions between two populations might be responsible for the discrepancy. Another possibility is that different genetic markers (SNPs and CNVs) might be linked with different disease status or clinical characteristics. Furthermore, the definition of age-at-onset of schizophrenia may also vary across studies. These diversities of clinical assessment also in some extent elucidated for the inconsistent findings across studies. Other clinical examinations or endophenotypes of schizophrenia need to be evaluated when further genetic association studies are implemented. The relationship between the genetic variants in the middle or 3'-end region of *NRXN1 *gene and schizophrenia would be verified in the future study.

## Conclusions

Our case-control association study suggested that *NRXN1 *gene may play a role in genetic susceptibility to schizophrenia. On the other hand, the different ethnic genetic structure of *NRXN1 *gene might influence the association of the polymorphisms with age-at-onset. Furthermore, our results also reinforce the need for the detailed LD mapping, CNV analysis of *NRXN1 *in different population or other neurodevelopmental disorders. The biological effect of risk variants and haplotypes in the *NRXN1 *gene associated with schizophrenia, and their role in disease pathogenesis and clinical characteristics, needs to be explored.

## List of abbreviations

*NRXN1*: neurexin-1; SNP: single nucleotide polymorphism; CNV: copy number variations; NLGN: neuroligins; DSM-IV: Diagnosis and statistic manual of mental disorders, 4^th ^edition; PCR: polymerase chain reactions; GPC: genetic power calculator; LD: linkage disequilibrium; OR: odds ratio; ASD: autism spectrum disorder; CDCV: common disease/common variant.

## Competing interests

The authors declare that they have no competing interests.

## Authors' contributions

DZ and WY participated in the design of the study and made final approval of the version to be published. WY and YY were involved in drafting the manuscript and data analysis. WY, YY, YZ, TL, XH, LW, and YR carried out the molecular genetic examination. WY, YY, YZ, XH, LW, and LL conducted sample selection and data management. All authors read and approved the final manuscript.
